# Fifteen Years of PNPLA3: Transforming Hepatology Through Human Genetics

**DOI:** 10.1111/liv.70240

**Published:** 2025-08-01

**Authors:** Stefano Romeo, Luca Valenti

**Affiliations:** ^1^ Department of Molecular and Clinical Medicine Institute of Medicine, Sahlgrenska Academy, Wallenberg Laboratory, University of Gothenburg Gothenburg Sweden; ^2^ Department of Medicine Karolinska University Stockholm Sweden; ^3^ Clinical Nutrition Unit Department of Medical and Surgical Science, University Magna Graecia Catanzaro Italy; ^4^ Department of Pathophysiology and Transplantation Università Degli Studi di Milano Milan Italy; ^5^ Precision Medicine and Biological Resource Center Fondazione IRCCS ca' Granda Ospedale Maggiore Policlinico Milano Milan Italy

**Keywords:** genetics, MASLD, PNPLA3, steatosis

## Abstract

Steatotic liver disease (SLD), caused by excess lipid accumulation in hepatocytes, is now a leading global liver condition triggered by metabolic dysfunction, alcohol, toxins and heritable factors. The main genetic determinant is the common *PNPLA3* p.I148M variant, which explains a substantial portion of SLD interindividual and interethnic variability, up to a quarter of liability to cirrhosis and liver cancer in the general population. Discovered in 2008 through genome‐wide association studies, this variant increases the risk of liver steatosis, inflammation, fibrosis and the risk of complications as hepatocellular carcinoma. Carriers, particularly homozygotes, show higher susceptibility to liver damage, with the variant interacting with environmental factors such as increased adiposity, diet, insulin resistance and female sex. Experimental models indicate a complex mechanistic impact, involving lipid transfer and retinoid metabolism. Phase 1 clinical trials exploring PNPLA3 inhibitors, such as siRNA and antisense oligonucleotides, are promising, demonstrating up to 40% reductions in hepatic fat and inflammation in homozygous carriers. These advances suggest that targeting PNPLA3 can pave the way for precision medicine in hepatology. Overall, the discovery of PNPLA3's role has significantly enhanced understanding of liver steatosis, steatohepatitis and potential therapeutic interventions, highlighting its importance as a therapeutic target for SLD.

**Summary**

- Steatotic liver disease is defined by lipid accumulation in the liver, with the *PNPLA3* p.I148M genetic variant being a key factor affecting individual and ethnic susceptibility to the condition and its complications like cirrhosis and liver cancer.
- The PNPLA3 variant heightens the risk of liver damage, especially in homozygous carriers, interacting with factors such as obesity, diet, insulin resistance and sex, influencing the progression of liver steatosis, inflammation and fibrosis.
- Recent clinical trials targeting PNPLA3 with siRNA and antisense oligonucleotides have shown promising results, including significant reductions in liver fat and inflammation, highlighting the variant's potential as a therapeutic target and for advancing precision medicine in hepatology.

## 1 Introduction

Steatotic liver disease (SLD) is a liver dysfunction caused by excess lipid accumulation in hepatocytes, mainly in the form of triglycerides (TAG) and has become the leading cause of liver disease globally [1]. SLD is indeed triggered either by metabolic dysfunction (MASLD: metabolic dysfunction associated SLD), excess alcohol, hepatotoxins and inherited factors [1, 2]. SLD is a highly heterogeneous condition, associated with an increased risk of liver‐related events and extra‐hepatic mortality. Due to the increasing incidence of MASLD, there is an urgent medical need for risk stratification biomarkers and for treatments to halt liver disease progression [3]. SLD has a large heritable component contributing to this clinical heterogeneity [4]. The common rs738409 C > G inherited variant encoding for an isoleucine to methionine substitution at position 148 of the patatin‐like phospholipase domain containing 3 protein (*PNPLA3* p.I148M variant) has a large effect on the risk of SLD [5, 6]. The p.I148M variant accounts for the largest fraction of SLD genetic variability, explaining about a quarter of liver disease liability in the European population and a large fraction of interethnic variability in SLD development [4, 5, 6]. The prevalence of the variant is higher in Native American and East Asian populations and lower in African vs. European populations [6]. The p.I148M variant was fixed in archaic humans, possibly to enhance cold adaptation, but segregated neutrally in the last 10 000 years [7].

The first early phase clinical trials examining the impact of *PNPLA3* inhibitors in carriers of the p.I148M variant have recently been published [8, 9]. In a Phase 1 study, silencing of *PNPLA3* by a liver‐targeted small interfering RNA (siRNA) was well tolerated and resulted in a dose‐dependent reduction of liver triglyceride content, up to 40% on the maximal dose [8]. Interestingly, the effect was present in homozygous, but not in heterozygous, carriers [8]. In keeping, in another Phase 1 clinical trial, *PNPLA3* downregulation by liver‐targeted antisense oligonucleotides (ASO) resulted in a dose‐dependent reduction of hepatic fat and inflammation, specifically in homozygous carriers [9]. These studies culminated a whole line of research after 15 years from the discovery of *PNPLA3*, representing the first proof of concept in humans of precision medicine using an inborn common genetic variant in a complex trait, namely SLD [10]. Here, we retraced the milestones of this story from the beginning.

**FIGURE 1:**
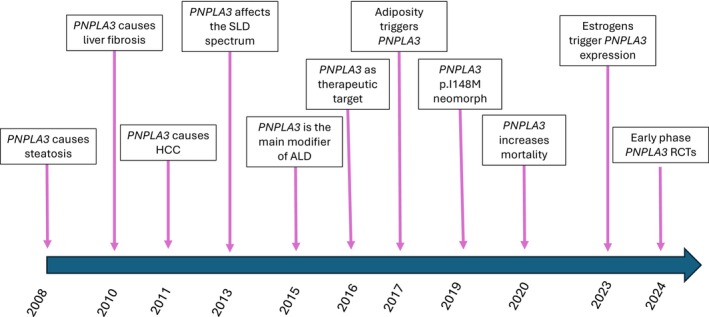
Milestones in the PNPLA3 research field.

In this short narrative review, we summarised the milestones from the discovery of the *PNPLA3* variant as the major inherited determinant of hepatic fat to the first proof of concept that downregulation in humans is effective (Figure 1). This is the introductory paper of a special issue on PNPLA3, where we have gathered the scientists that have contributed most to this topic to present all the facets of this story. Here, we presented our own personal view and for reasons of space we could not accommodate all the papers published on PNPLA3, which are now more than 1400. Since clinical aspects are covered in the special issue reviews, we prioritised key mechanistic and translational milestones.

## 2 Identification of *PNPLA3* as Genetic Determinant of Liver Triglyceride Content

In 2008, relatively late compared to other fields of medicine, the first exome‐wide association study examining the association of liver TAG content with protein‐coding variants in the Dallas Hart Study (DHS) identified only one genetic variant exceeding the *p* value adjusted for multiple testing comparison and therefore likely to be a true finding. This variant was the *PNPLA3* p.I148M [11].

If we take a closer look at the study strategy, Helen Hobbs and Jonathan Cohen were pioneers in foreseeing several key aspects of human genetics of common traits. For the first, they restricted the number of genetic variants using only those resulting in an amino acid change at a protein level to reduce multiple testing and the possibility of false positives. This strategy was also used because these variants are those more likely to cause changes in protein function and therefore result in phenotypic changes. Second, the DHS is one of the first highly phenotyped cohorts used in genome‐wide studies; its use of precise measurements—specifically magnetic spectroscopy to quantify liver triglyceride content—massively boosted statistical power by stripping out noise. Third, taking advantage of ethnic diversity in the genome, the DHS was designed to be composed of three ethnic groups, including Europeans, African Americans and Hispanics, becoming one of the first trans‐ancestry genomic studies. Exploiting differences in the frequency of genetic variants among ethnic groups contributes to the increase in the signal at any locus. Interestingly, the study also detected a low‐frequency variant that was present only in African Americans (rs6006460 encoding for p.S453I), where carriers of the minor allele had lower hepatic TAG levels. The presence of another common variant pointed out the fact that the *PNPLA3* signal was real [11].

However, when looking at liver phenotypes, there was an association with increased alanine aminotransferases, a biomarker of liver damage, only in Hispanics and not in the other ethnic groups, leaving questions on the relevance of the gene variant on liver disease.

## 3 Relevance of *PNPLA3* p.I148M in Liver Disease

It soon became clear that the p.I148M variant was not only associated with steatosis, but also with the progression to metabolic steatohepatitis (MASH), and liver fibrosis, the main determinant of liver events [12]. It has also recently been reported that liver fat accumulation displays a distinct pattern in carriers of the *PNPLA3* variant [13].

However, at that time it was not clear whether liver TAG accumulation was per se deleterious, contributing to liver inflammation and fibrosis, or an innocent bystander in disease pathogenesis [14]. Notably, the first large multicentric study examining the effect of the association of the variant in individuals with liver histology confirmed that the variant was associated with the whole spectrum of the condition and that it was preferentially inherited by children with severe MASLD [12]. This study also showed for the first time that in individuals homozygous for the variant there was an upregulation of inflammatory pathways, despite fat accumulation being paradoxically associated with reduced de novo lipogenesis [12].

Genetic epidemiological associations were then confirmed in a meta‐analysis, hinting for the first time at a larger impact of the variant in women [15]. Moreover, the variant was associated with increased liver‐related and overall mortality in general adult populations characterised by high prevalence of obesity in the NHANES cohort [16].

Serendipitously, a genome‐wide association study (GWAS) examining individuals with liver cirrhosis due to alcohol consumption identified the minor allele of the same variant in *PNPLA3* as the strongest genetic determinant of this late liver complication in people with alcoholic SLD (ALD) [17]. At that time, non‐alcoholic and alcoholic SLD were considered two different diseases. This seminal GWAS provided the first proof of concept that these diseases have similar molecular mechanisms largely mediated by the excess in liver fat. A large impact of the p.I148M variant on liver damage was also observed in other liver conditions where hepatic fat accumulation plays a key role, such as SLD in children and in lean adults, and in those with chronic hepatitis C and hemochromatosis [18, 19, 20].

Next, it turned out that the p.I148M variant predisposes to hepatocellular carcinoma (HCC), irrespective of the drivers of SLD [21, 22, 23]. Notably, HCC liability was particularly evident in people homozygous for the variant.

*PNPLA3* genotyping is therefore now being included in the clinical evaluation of people with SLD [24].

## 4 Interaction of *PNPLA3* With the Environment

It was next demonstrated that the p.I148M variant interacts with excess body weight and insulin resistance to determine SLD [25]. This was shown for the first time by a study on morbidly obese individuals where the genetic variant was associated with transaminases and then confirmed by a systematic and definitive analysis of the interaction in population cohorts [26, 27]. Qualitative aspects of the diet, and specifically the content in carbohydrates, saturated fatty acids, red meats and the low content in vegetables, modify *PNPLA3* variant expression [25, 28].

Subsequent studies showed that carriers of the variants have different responses to lifestyle approaches based on diet and physical exercise. Some studies also reported a reduced benefit of treatments acting on hepatic lipid metabolism, such as statins and omega 3 fatty acids on liver damage in carriers of *PNPLA3* p.I14M [25, 29, 30]. Interestingly, carriers of the *PNPLA3* variant were more likely to have toxicity after the use of other drugs such as insulin, chemotherapy and non‐steroidal anti‐inflammatory drugs [31].

Another important finding was the demonstration of an interaction between the risk allele and sex where the deleterious effect of the p.I148M variant is larger in women than in men due to the hormonal environment (see later why) [32, 33].

## 5 Metabolic Effect of *PNPLA3*

Given the large impact on lipid accumulation in hepatocytes, the *PNPLA3* variant has several pleiotropic effects on metabolic traits and conditions affecting the liver and other organs. Indeed, in large population studies, carriage of the *PNPLA3* variant was associated with a modest increase in diabetes risk [34, 35]. This is not a pleiotropic effect of the variant itself but rather reflects the degree of hepatic fat accumulation, similar to what is seen with other SLD‐associated genetic variants. The increased risk appears to be mediated by liver damage, likely secondary to either reduced insulin clearance due to steatosis or a direct effect on hepatic insulin resistance driven by lipotoxicity [34].

Similarly, the variant has been associated with a small increase in the risk of kidney damage in people with metabolic risk factors, which seems proportional to the impact on liver steatosis, although a direct effect on kidney cells cannot be ruled out [36].

On the other hand, the *PNPLA3* variant has been linked with a small impairment of lipid secretion, not fully compensated by the higher liver content, in people with obesity. Therefore, carriers tend to have slightly lower circulating triglycerides but notably also non‐HDL cholesterol, mitigating the predisposition to cardiovascular disease caused by the increased diabetes [37].

## 6 Enzymatic Activity of PNPLA3

The PNPLA3 protein is expressed on lipid droplets in hepatocytes and other cell types, where it is induced by insulin and oestrogens [32, 38]. However, the function of wild‐type and p.I148M PNPLA3 protein remains, for some aspect, controversial. Purified protein incubated with a radiolabelled radioactive substrate has a TAG hydrolase activity releasing free fatty acids from the backbone of glycerol. An important aspect to consider is that the enzymatic activity is preferentially directed against mono‐ and polyunsaturated as opposed to saturated fatty acids. The amino acid substitution p.I148M abolishes this enzymatic activity [39, 40].

The mechanism underlying the phenotypic expression of the variants has been studied in experimental models with conflicting results [41]. Knockout of *Pnpla3* in mice does not have any evident phenotype irrespective of the diet. One thing to consider is that there is a relatively low homology of Pnpla3 between mouse and human, where the protein in mice is much shorter [42]. Knock‐in mice showed a mild increase in liver TAG content only on a high sucrose diet with metabolic refeeding and specifically in female mice [42]. A lipidomic study in genetically modified mice suggested the wild‐type PNPLA3 protein promotes the transfer of polyunsaturated fatty acids from triglycerides to phospholipids in hepatic lipid droplets (trans‐acylase activity) [43]. In this model, the mutant protein behaves as a gain of function concerning intracellular lipid accumulation [42]. However, polyunsaturated fatty acids are a small amount compared to mono and saturated fatty acids, and therefore, the large observed effect of the PNPLA3 variant is likely deriving from the absence of triglyceride hydrolysis on monounsaturated due to the sequestering of CGI‐58/ABHD5, an essential cofactor for patatin‐like lipases. According to this model, the PNPLA3 p.I148M protein variant behaves as a gain of new function (neomorph) that accumulates on lipid droplets, leading to the inhibition of ATGL/PNPLA2, the main TAG hydrolase [44, 45].

*PNPLA3* is also highly expressed in hepatic stellate and in cells from the retina [46, 47]. These cells have in common the ability to store derivatives of vitamin A, namely retinoids. This similarity prompted the study of the role of PNPLA3 in hepatic stellate cells (HSC) against retinyl esters, which resulted in reduced carriers, thereby altering HSC phenotype and fibrogenic properties [46, 47, 48].

Whether this impaired activity of PNPLA3 in HSC has a major clinical role in determining hepatic inflammation and predisposition to HCC independent of hepatic fat accumulation remains to be determined [48]. Experimental models and evaluation of the impact of donor/recipient after liver transplantation may help to address these questions [41, 49].

To summarise, the *PNPLA3* p.I148M variant determines hepatic fat accumulation and lipotoxicity through a gain of new function by inhibiting PNPLA2 and LD remodelling in hepatocytes. The extent to which the loss of enzymatic activity and the impairment of retinol metabolism in HSC that are also associated with the variant contribute to the liver phenotype remains to be determined.

## 7 Beneficial Effect of Downregulation of PNPLA3 Against SLD

After the demonstration that PNPLA3 p.I148M protein accumulates on LDs due to resistance to ubiquitylation [50] and that this is necessary for the phenotypic expression of steatosis, we hypothesised that targeting mutant PNPLA3 for silencing may represent a therapeutic approach in carriers [51].

By a human genetics approach through the sequencing of patients with early onset severe disease and controls, we found that another variant in *PNPLA3*, namely rs2294918 encoding p.K434E, which in Europeans segregates with the 148M allele, is required for its phenotypic expression [52]. Indeed, we showed that in the rare cases when this is not true the impact of p.I148M was blunted [52]. On the other hand, the same variant increases the protective association of wild‐type PNPLA3 on liver damage. We showed that this p.K453E variant does not impact TAG hydrolase activity but is associated with increased protein expression in the human liver. This is consistent with a model where the accumulation of the enzymatically inactive p.I148M inhibits PNPLA2, leading to liver disease, whereas higher levels of wild‐type enzymatically active PNPLA3 may even be protective.

These data supported for the first time the notion of *PNPLA3* as a therapeutic target in homozygotes carrying the p.I148M to rescue the inhibition on PNPLA2. On the other hand, they also suggest that silencing PNPLA3 in heterozygotes may have unpredictable results because likely the enzymatic activity of the wild‐type protein still plays a beneficial role. It also leaves open the possibility that even in homozygotes, PNPLA3 silencing in the absence of a replacement of the missing enzymatic activity may not fully restore the risk of liver disease to the baseline. Additional studies are necessary to examine the impact of long‐term suppression of PNPLA3 p.I148M protein on liver fibrosis and on the development of hepatic and extra‐hepatic events, both in homozygous and heterozygous carriers with SLD spectrum disorders.

## 8 Conclusions and Remarks

**FIGURE 2:**
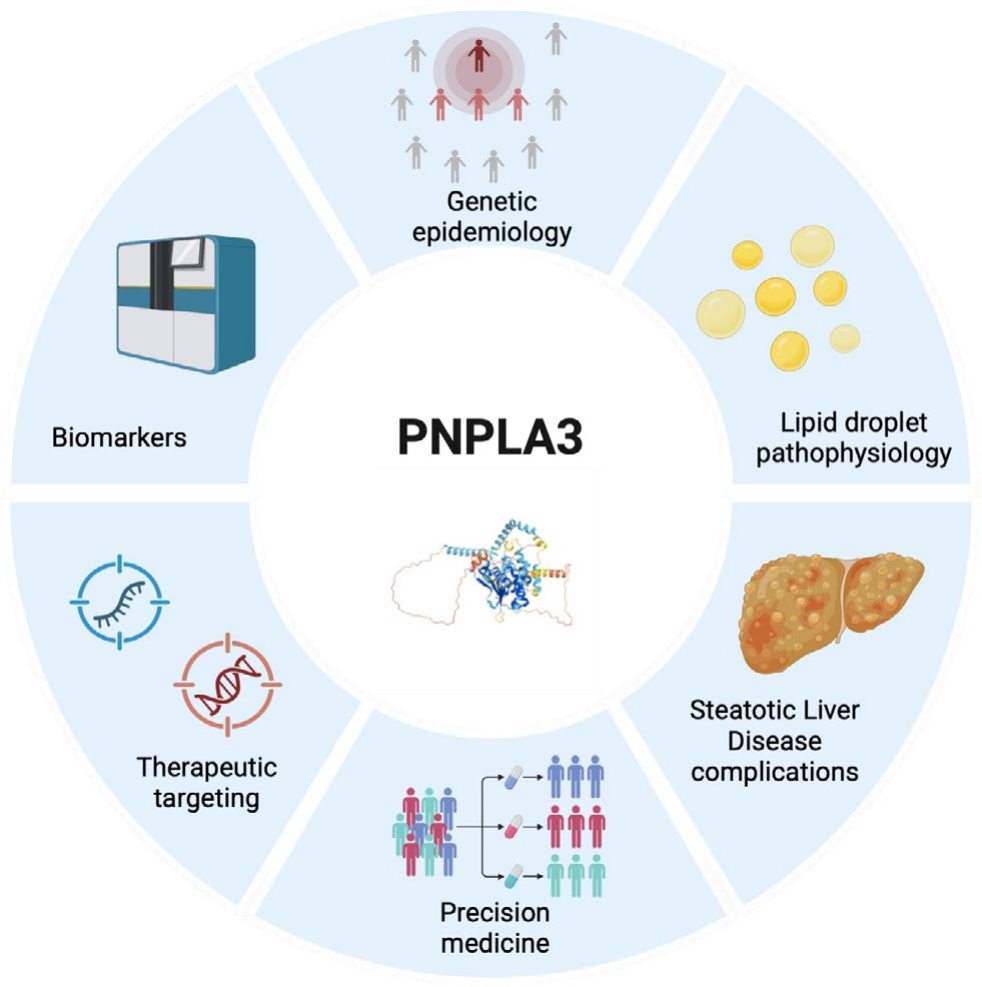
Impact of PNPLA3 research in hepatology.

All in all, the discovery of *PNPLA3* variation as a main determinant of liver disease and the research field it has opened has brought about several innovations in liver research (Figure 2). These range from the identification of the causes of liver disease, with a focus on hepatic fat accumulation as not only the trigger‐independent hallmark but the driver of progression of SLD, of the metabolic and systemic consequences of steatosis, non‐invasive biomarkers discovery and therapeutic targeting. These novel therapeutic approaches may also be relevant for the management of other cardiometabolic and kidney diseases.

Several modalities targeting PNPLA3 for silencing are already under clinical development, which may enable the first precision medicine approach in hepatology tackling the roots of liver disease [10].

## Conflicts of Interest

Authors do not report COI relevant to the present manuscript. L.V. reports speaking fees from: Viatris, Novo Nordisk, GSK; consulting for: Novo Nordisk, Pfizer, Boehringer Ingelheim, Resalis, GSK, Almac.

## Data Availability

The authors have nothing to report.

## References

[1] M. Israelsen , S. Francque , E. A. Tsochatzis , and A. Krag , “Steatotic Liver Disease,” Lancet 404 (2024): 1761–1778. 39488409 10.1016/S0140-6736(24)01811-7

[2] M. E. Rinella , J. V. Lazarus , V. Ratziu , et al., “A Multisociety Delphi Consensus Statement on New Fatty Liver Disease Nomenclature,” Journal of Hepatology 79 (2023): 1542–1556. 37364790 10.1016/j.jhep.2023.06.003

[3] European Association for the Study of the Liver (EASL) , European Association for the Study of Diabetes (EASD) , European Association for the Study of Obesity (EASO) , and European Association for the Study of the Liver (EASL) , “EASL‐EASD‐EASO Clinical Practice Guidelines on the Management of Metabolic Dysfunction‐Associated Steatotic Liver Disease (MASLD),” Journal of Hepatology 81, no. 3 (2024): 492–542, 10.1016/j.jhep.2024.04.031. 38851997

[4] S. Sookoian , Y. Rotman , and L. Valenti , “Genetics of Metabolic Dysfunction‐Associated Steatotic Liver Disease: The State of the Art Update,” Clinical Gastroenterology and Hepatology 22 (2024): 2177–2187.e3. 39094912 10.1016/j.cgh.2024.05.052 PMC11512675

[5] O. Jamialahmadi , C. Bianco , S. Pelusi , S. Romeo , and L. Valenti , “Reply to: Polygenic Risk Score: A Promising Predictor for Hepatocellular Carcinoma in the Population With Non‐Alcoholic Fatty Liver Disease,” Journal of Hepatology 74 (2021): 1494–1496. 33676949 10.1016/j.jhep.2021.02.030

[6] J. Kozlitina and S. Sookoian , “Global Epidemiological Impact of PNPLA3 I148M on Liver Disease,” Liver International 45 (2025): e16123. 39373119 10.1111/liv.16123 PMC11815610

[7] A. Geier , J. Trost , K. Wang , C. Schmid , M. Krawczyk , and S. Schiffels , “PNPLA3 Fatty Liver Allele Was Fixed in Neanderthals and Segregates Neutrally in Humans,” Gut 73 (2024): 1008–1014. 38458749 10.1136/gutjnl-2023-331594

[8] E. Fabbrini , B. Rady , A. Koshkina , et al., “Phase 1 Trials of PNPLA3 siRNA in I148M Homozygous Patients With MAFLD,” New England Journal of Medicine 391 (2024): 475–476. 39083780 10.1056/NEJMc2402341

[9] J. Armisen , M. Rauschecker , J. Sarv , et al., “AZD2693, a PNPLA3 Antisense Oligonucleotide, for the Treatment of MASH in 148M Homozygous Participants: Two Randomized Phase I Trials,” Journal of Hepatology 83, no. 1 (2025): 31–42, 10.1016/j.jhep.2024.12.046. 39798707

[10] D. Lindén , G. Tesz , and R. Loomba , “Targeting PNPLA3 to Treat MASH and MASH Related Fibrosis and Cirrhosis,” Liver International 45 (2025): e16186. 39605307 10.1111/liv.16186 PMC11907219

[11] S. Romeo , J. Kozlitina , C. Xing , et al., “Genetic Variation in PNPLA3 Confers Susceptibility to Nonalcoholic Fatty Liver Disease,” Nature Genetics 40 (2008): 1461–1465. 18820647 10.1038/ng.257 PMC2597056

[12] L. Valenti , A. Al‐Serri , A. K. Daly , et al., “Homozygosity for the Patatin‐Like Phospholipase‐3/Adiponutrin I148M Polymorphism Influences Liver Fibrosis in Patients With Nonalcoholic Fatty Liver Disease,” Hepatology 51 (2010): 1209–1217. 20373368 10.1002/hep.23622

[13] Y. Chen , B. P. M. Laevens , T. Lemainque , et al., “Deep Learning Reveals Liver MRI Features Associated With PNPLA3 I148M in Steatotic Liver Disease,” Liver International 45 (2025): e70164. 40478199 10.1111/liv.70164 PMC12143367

[14] C. P. Day and O. F. James , “Steatohepatitis: A Tale of Two “Hits”?,” Gastroenterology 114 (1998): 842–845. 9547102 10.1016/s0016-5085(98)70599-2

[15] S. Sookoian and C. J. Pirola , “Meta‐Analysis of the Influence of I148M Variant of Patatin‐Like Phospholipase Domain Containing 3 Gene (PNPLA3) on the Susceptibility and Histological Severity of Nonalcoholic Fatty Liver Disease,” Hepatology 53 (2011): 1883–1894. 21381068 10.1002/hep.24283

[16] A. Unalp‐Arida and C. E. Ruhl , “Patatin‐Like Phospholipase Domain‐Containing Protein 3 I148M and Liver Fat and Fibrosis Scores Predict Liver Disease Mortality in the U.S. Population,” Hepatology 71 (2020): 820–834. 31705824 10.1002/hep.31032

[17] S. Buch , F. Stickel , E. Trépo , et al., “A Genome‐Wide Association Study Confirms PNPLA3 and Identifies TM6SF2 and MBOAT7 as Risk Loci for Alcohol‐Related Cirrhosis,” Nature Genetics 47 (2015): 1443–1448. 26482880 10.1038/ng.3417

[18] L. Valenti , A. Alisi , E. Galmozzi , et al., “I148M Patatin‐Like Phospholipase Domain‐Containing 3 Gene Variant and Severity of Pediatric Nonalcoholic Fatty Liver Disease,” Hepatology 52 (2010): 1274–1280. 20648474 10.1002/hep.23823

[19] P. Dongiovanni , B. Donati , R. Fares , et al., “PNPLA3 I148M Polymorphism and Progressive Liver Disease,” World Journal of Gastroenterology 19 (2013): 6969–6978. 24222941 10.3748/wjg.v19.i41.6969 PMC3819533

[20] Y. Seko , H. Lin , V. W.‐S. Wong , and T. Okanoue , “Impact of PNPLA3 in Lean Individuals and in Cryptogenic Steatotic Liver Disease,” Liver International 45 (2025): e16164. 39540675 10.1111/liv.16164

[21] L. Valenti , M. Rumi , E. Galmozzi , et al., “Patatin‐Like Phospholipase Domain‐Containing 3 I148M Polymorphism, Steatosis, and Liver Damage in Chronic Hepatitis C,” Hepatology 53 (2011): 791–799. 21319195 10.1002/hep.24123

[22] E. Trépo , P. Nahon , G. Bontempi , et al., “Association Between the PNPLA3 (rs738409 C>G) Variant and Hepatocellular Carcinoma: Evidence From a Meta‐Analysis of Individual Participant Data,” Hepatology 59 (2014): 2170–2177. 24114809 10.1002/hep.26767

[23] F. Tavaglione , G. Pennisi , and S. Pelusi , “PNPLA3 I148M and Hepatocellular Carcinoma,” Liver International 45 (2025): e70051. 40029157 10.1111/liv.70051

[24] L. Ronzoni , S. Pelusi , V. Moretti , et al., “Diagnostic Uptake of Targeted Sequencing in Adults With Steatotic Liver Disease and a Suspected Genetic Contribution,” Liver International 45 (2025): e70010. 39945383 10.1111/liv.70010 PMC11822878

[25] E. K. Speliotes and C. V. Schneider , “PNPLA3 I148M Interacts With Environmental Triggers to Cause Human Disease,” Liver International 45 (2025): e16106. 39559944 10.1111/liv.16106 PMC11815600

[26] S. Romeo , F. Sentinelli , S. Dash , et al., “Morbid Obesity Exposes the Association Between PNPLA3 I148M (rs738409) and Indices of Hepatic Injury in Individuals of European Descent,” International Journal of Obesity 34 (2010): 190–194. 19844213 10.1038/ijo.2009.216

[27] S. Stender , J. Kozlitina , B. G. Nordestgaard , A. Tybjærg‐Hansen , H. H. Hobbs , and J. C. Cohen , “Adiposity Amplifies the Genetic Risk of Fatty Liver Disease Conferred by Multiple Loci,” Nature Genetics 49 (2017): 842–847. 28436986 10.1038/ng.3855 PMC5562020

[28] V. Nobili , D. Liccardo , G. Bedogni , et al., “Influence of Dietary Pattern, Physical Activity, and I148M PNPLA3 on Steatosis Severity in At‐Risk Adolescents,” Genes & Nutrition 9 (2014): 392. 24627307 10.1007/s12263-014-0392-8 PMC4026440

[29] V. Nobili , G. Bedogni , B. Donati , A. Alisi , and L. Valenti , “The I148M Variant of PNPLA3 Reduces the Response to Docosahexaenoic Acid in Children With Non‐Alcoholic Fatty Liver Disease,” Journal of Medicinal Food 16 (2013): 957–960. 24074360 10.1089/jmf.2013.0043

[30] P. Dongiovanni , S. Petta , V. Mannisto , et al., “Statin Use and Non‐Alcoholic Steatohepatitis in at Risk Individuals,” Journal of Hepatology 63 (2015): 705–712. 25980762 10.1016/j.jhep.2015.05.006

[31] D. Diogo , C. Tian , C. S. Franklin , et al., “Phenome‐Wide Association Studies Across Large Population Cohorts Support Drug Target Validation,” Nature Communications 9 (2018): 4285. 10.1038/s41467-018-06540-3 PMC6191429 30327483

[32] A. Cherubini , M. Ostadreza , O. Jamialahmadi , et al., “Interaction Between Estrogen Receptor‐α and PNPLA3 p.I148M Variant Drives Fatty Liver Disease Susceptibility in Women,” Nature Medicine 29 (2023): 2643–2655. 10.1038/s41591-023-02553-8 PMC10579099 37749332

[33] A. Cherubini , C. Rosso , and S. Della Torre , “Sex‐Specific Effects of PNPLA3 I148M,” Liver International 45 (2025): e16088. 39262132 10.1111/liv.16088 PMC11815604

[34] P. Dongiovanni , S. Stender , A. Pietrelli , et al., “Causal Relationship of Hepatic Fat With Liver Damage and Insulin Resistance in Nonalcoholic Fatty Liver,” Journal of Internal Medicine 283 (2018): 356–370. 29280273 10.1111/joim.12719 PMC5900872

[35] H. Yki‐Järvinen and P. K. Luukkonen , “Function of PNPLA3 I148M‐Lessons From In Vivo Studies in Humans,” Liver International 45 (2025): e70047. 40052746 10.1111/liv.70047

[36] A. Mantovani and G. Targher , “PNPLA3 Variation and Kidney Disease,” Liver International 45 (2025): e16010. 38873992 10.1111/liv.16010

[37] K. Wijarnpreecha , M. Scribani , P. Raymond , et al., “PNPLA3 Gene Polymorphism and Overall and Cardiovascular Mortality in the United States,” Journal of Gastroenterology and Hepatology 35 (2020): 1789–1794. 32220085 10.1111/jgh.15045

[38] Y. Huang , S. He , J. Z. Li , et al., “A Feed‐Forward Loop Amplifies Nutritional Regulation of PNPLA3,” Proceedings of the National Academy of Sciences of the United States of America 107 (2010): 7892–7897. 20385813 10.1073/pnas.1003585107 PMC2867902

[39] S. He , C. McPhaul , J. Z. Li , et al., “A Sequence Variation (I148M) in PNPLA3 Associated With Nonalcoholic Fatty Liver Disease Disrupts Triglyceride Hydrolysis,” Journal of Biological Chemistry 285 (2010): 6706–6715. 20034933 10.1074/jbc.M109.064501 PMC2825465

[40] P. Pingitore , C. Pirazzi , R. M. Mancina , et al., “Recombinant PNPLA3 Protein Shows Triglyceride Hydrolase Activity and Its I148M Mutation Results in Loss of Function,” Biochimica et Biophysica Acta 1841 (2014): 574–580. 24369119 10.1016/j.bbalip.2013.12.006

[41] A. Ramandi , A.‐M. Diehl , A. J. Sanyal , and Y. P. de Jong , “Experimental Models to Investigate PNPLA3 in Liver Steatosis,” Liver International 45 (2025): e70091. 40231787 10.1111/liv.70091 PMC12147532

[42] E. Smagris , S. BasuRay , J. Li , et al., “Pnpla3I148M Knockin Mice Accumulate PNPLA3 on Lipid Droplets and Develop Hepatic Steatosis,” Hepatology 61 (2015): 108–118. 24917523 10.1002/hep.27242 PMC4262735

[43] M. A. Mitsche , H. H. Hobbs , and J. C. Cohen , “Patatin‐Like Phospholipase Domain–Containing Protein 3 Promotes Transfer of Essential Fatty Acids From Triglycerides to Phospholipids in Hepatic Lipid Droplets,” Journal of Biological Chemistry 293 (2018): 6958–6968. 29555681 10.1074/jbc.RA118.002333 PMC5936833

[44] Y. Wang , N. Kory , S. BasuRay , J. C. Cohen , and H. H. Hobbs , “PNPLA3, CGI‐58, and Inhibition of Hepatic Triglyceride Hydrolysis in Mice,” Hepatology 69 (2019): 2427–2441. 30802989 10.1002/hep.30583 PMC6563103

[45] Y. Wang , S. Hong , H. Hudson , et al., “PNPLA3(148M) is a Gain‐Of‐Function Mutation That Promotes Hepatic Steatosis by Inhibiting ATGL‐Mediated Triglyceride Hydrolysis,” Journal of Hepatology 82 (2025): 871–881. 39550037 10.1016/j.jhep.2024.10.048 PMC12164368

[46] C. Pirazzi , L. Valenti , B. M. Motta , et al., “PNPLA3 Has Retinyl‐Palmitate Lipase Activity in Human Hepatic Stellate Cells,” Human Molecular Genetics 23 (2014): 4077–4085. 24670599 10.1093/hmg/ddu121 PMC4082369

[47] P. Pingitore , P. Dongiovanni , B. M. Motta , et al., “PNPLA3 Overexpression Results in Reduction of Proteins Predisposing to Fibrosis,” Human Molecular Genetics 25 (2016): 5212–5222. 27742777 10.1093/hmg/ddw341 PMC5886043

[48] M. Castanho Martins , E. D. Dixon , G. Lupo , T. Claudel , M. Trauner , and K. Rombouts , “Role of PNPLA3 in Hepatic Stellate Cells and Hepatic Cellular Crosstalk,” Liver International 45 (2025): e16117. 39394864 10.1111/liv.16117 PMC11891384

[49] V. Liukkonen , M. Semenova , K. Hyvärinen , et al., “Genetic Risk Factors for Steatotic Liver Disease After Liver Transplantation,” Liver International 45 (2025): e70067. 40087975 10.1111/liv.70067

[50] S. BasuRay , Y. Wang , E. Smagris , J. C. Cohen , and H. H. Hobbs , “Accumulation of PNPLA3 on Lipid Droplets Is the Basis of Associated Hepatic Steatosis,” Proceedings of the National Academy of Sciences of the United States of America 116 (2019): 9521–9526. 31019090 10.1073/pnas.1901974116 PMC6511016

[51] L. Valenti and P. Dongiovanni , “Mutant PNPLA3 I148M Protein as Pharmacological Target for Liver Disease,” Hepatology 66 (2017): 1026–1028. 28586091 10.1002/hep.29298

[52] B. Donati , B. M. Motta , P. Pingitore , et al., “The rs2294918 E434K Variant Modulates Patatin‐Like Phospholipase Domain‐Containing 3 Expression and Liver Damage,” Hepatology 63 (2016): 787–798. 26605757 10.1002/hep.28370

